# Endosomal binding kinetics of Eps15 and Hrs specifically regulate the degradation of RTKs

**DOI:** 10.1038/s41598-017-17320-2

**Published:** 2017-12-21

**Authors:** Linda Hofstad Haugen, Frode Miltzow Skjeldal, Trygve Bergeland, Oddmund Bakke

**Affiliations:** 1 Department of Biosciences, Centre of Immune Regulation, University of Oslo, Oslo, Norway; 2 Present Address: Kappa Bioscience AS, Oslo, Norway

## Abstract

Activation of EGF-R and PDGF-R triggers autophosphorylation and the recruitment of Eps15 and Hrs. These two endosomal proteins are important for specific receptor sorting. Hrs is recruiting ubiquitinated receptors to early endosomes to further facilitate degradation through the ESCRT complex. Upon receptor activation Hrs becomes phosphorylated and is relocated to the cytosol, important for receptor degradation. In this work we have studied the endosomal binding dynamics of Eps15 and Hrs upon EGF-R and PDGF-R stimulation. By analysing the fluorescence intensity on single endosomes after ligand stimulation we measured a time-specific decrease in the endosomal fluorescence level of Eps15-GFP and Hrs-YFP. Through FRAP experiments we could further register a specific change in the endosomal-membrane to cytosol binding properties of Eps15-GFP and Hrs-YFP. This specific change in membrane fractions proved to be a redistribution of the immobile fraction, which was not shown for the phosphorylation deficient mutants. We here describe a mechanism that can explain the previously observed relocation of Hrs from the endosomes to cytosol after EGF stimulation and show that Eps15 follows a similar mechanism. Moreover, this specific redistribution of the endosomal protein binding dynamics proved to be of major importance for receptor degradation.

## Introduction

Receptor tyrosine kinases (RTK) play an important role in the control of fundamental cellular processes, including the cell cycle, cell migration, cell metabolism and survival, cell proliferation and differentiation^1,2^. Binding of ligand is the activation signal for all the RTKs, which triggers trans-autophosphorylation of the receptor. This step is crucial for RTK dependent activation and recruitment of a variety of signalling proteins. Binding of ligand leads to ubiquitination of the receptor and recruitment of Hepatocyte growth factor-regulated tyrosine kinase substrate (Hrs) and Epidermal growth factor receptor pathway substrate 15 (Eps15). This process targets RTKs to the lumen of multivesicular bodies (MVBs) for lysosomal degradation^3^. Sorting of membrane receptors into MVBs is orchestrated by the sequential recruitment of members of the endosomal-sorting complex required for transport (ESCRT complex) (for review see^3^).

Epidermal growth factor receptor substrate 15 (Eps15) is an adaptor protein important for endocytosis^4^. The N-terminal Eps15 homology (EH) domains bind NPF motifs on a variety of other endocytic adaptor proteins. The central coiled-coil domain mediates Eps15 oligomerization and binding to other proteins including Hrs. The DPF domain of Eps15 binds to adaptor protein-2 (AP-2) and is important in the formation of clathrin-coated vesicles (CCV)^4,5^. In the C-terminus of Eps15 the two ubiquitin interacting motifs (UIM domains) are located. Eps15 has been reported to bind directly to ubiquitinated EGF-R through these UIM domains^6^. Activation of EGF-R triggers both monoubiquitination and phosphorylation of Eps15^7,8^.

Hrs is a 115-kDa multidomain coat protein that binds to the endosomal membrane either through the FYVE- (Fab-1, YGL023, Vps27, and EEA1) or the coiled-coiled domain^9–11^. Hrs recognizes ubiquitinated receptors through the ubiquitin interacting motif (UIM), and together with signal-transduction adaptor-molecule (STAM) it acts as part of the sorting machinery for degradation via the ESCRT machinery^12,13^. Upon EGF-R activation, Hrs is tyrosine phosphorylated and monoubiquitinated^14,15^.

In this study we have described a downstream effect of EGF-R ligand binding on the phosphorylation and membrane binding kinetics of Hrs and Eps15. To facilitate the analysis of the membrane binding kinetics on single endosomes we enlarged the endosomal size by transfecting the cells with the major histocompatibility complex class-II associated invariant chain (Ii) under the control of an inducible metallothionein promotor. Expression of Ii in model cell lines has been found to increase the early endosomal fusion rate and resulting in an enlargement of the endosomes^16–20^.

Eps15 and Hrs cycle between a membrane bound and a cytosolic state, and in this study we could measure that their binding properties change upon EGF and PDGF stimulation. We show that the membrane-to-cytosol cycling of both Eps15 and Hrs is dependent on their state of phosphorylation. For the first time we can document how RTK induced phosphorylation of Hrs and Eps15 regulates their endosomal binding kinetics. Receptor induced phosphorylation of Hrs and Eps15 stimulate a particular change in the equilibrium between the immobile and the mobile fractions. This redistribution changes the amounts of Hrs and Eps15 in the cytosol and can be accounted for by the release of Hrs or Eps15 from the immobile fraction. These results confirm and provide an explanation to previously published biochemical assays, which show a redistribution of the membrane bound fraction of Hrs to the cytosolic upon phosphorylation^21,22^. We can additionally show that the binding of Eps15 is regulated by phosphorylation in a similar manner. Our work provides an important mechanistic link between the receptor-induced phosphorylation of Hrs and Eps15 and their redistribution to cytosol. We furthermore show that this specific change in the endosomal binding kinetics is of major importance for the RTK degradation.

## Materials and Methods

### Constructs

cDNA encoding Ii-wt^23^ was subcloned into the pMep4 vector (Invitrogen). The pMep4 vector contains a metallothionein promoter and expression is induced by addition of 5 μM cadmium chloride (CdCl_2_) to the culture medium^24^. The fusion constructs; pEGFP-C2-Eps15, pEYFP-C1-Hrs and pEGFP-CtEEA1 (residue 1257–1411 of EEA1, here termed CtEEA1-GFP) has all been described earlier^25–27^.

Rab5–mCherry was made by amplifying canine Rab5 and mCherry separately by PCR using the following primers: for Rab5, 5′AGAGA**GGATCC**ATGGCTAATCGAGGAGCAAC-3′ (the BamHI site is in bold) and 5′-AGAGA**CTCGAG**TTAGTTACTACAACACTG-3′ (XhoI site is in bold); for mCherry, 5′-AGAGA**GGTACC** ATGGTGAGCAAGGGCGAGGAG-3′ (KpnI site) and 5′-AGAGA**GGATCC**CTTGTACAGCTCGTCCATGCC-3′ (BamHI site). mCherry was ligated to the N-terminus of Rab5 by the BamHI site and subsequently subcloned into the pcDNA3 vector (Invitrogen, Carlsbad, CA, US) at the KpnI and XhoI sites. All the restriction enzymes used in this cloning experiment is from New England Biolabs (NEB, Ipswich, UK).

To mutate tyrosine 850 to phenylalanine in pEGFP-C2-Eps15 a QuickChange Site-Directed Mutagenesis Kit (#200518, Stratagene, La Jola, CA, US) was used according to the protocol. Microsynth (Microsynth, Balgach, CH) supplied the primers and XL10-Gold Ultracompetent cells (#200314, Stratagene, La Jolla, CA, US) were used in the transformation of the plasmid. pEGFP-C2-Eps15 was sequenced at GATC (GACT Biotech AG, Constance, DE) and the mutation was confirmed. The same protocol as described above was used to mutate tyrosine 329 and 332 to phenylalanine in pEYFP-C1-Hrs.

### Cell lines

Human fibroblast (M1) cells were grown in complete medium: DMEM (Bio Whittaker MD, Walkersville, US) supplemented with 10% FCS (Integro B. V., Zaandam, NL), 2mM L-glutamine, 25U/ml penicillin and 25 μg/mL streptomycin (Bio Whittaker MD, Walkersville, US) in a 5% CO_2_ incubator with 37 °C. Cells stable transfected with Ii-pMep4 (Invitrogen, Carlsbad, CA, US) were grown in medium containing 0.15 mg/mL Hygromycin B (Saaven & Werner AB, Linhamn, SE). M1 cells stably expressing Hrs-YFP/Eps15-GFP and Ii-pMep4 were grown in complete medium containing 0.2 mg/mL G418 bisulphate (Duchefa, Haarlem, NL) and 0.15 mg/mL Hygromycin B (Saaven & Werner AB, Linhamn, SE). All the FRAP experiments were done on cells stably expressing Hrs-YFP or Eps15-GFP. Lipofectamine 2000 reagent (Invitrogen, Carlsbad, CA, US) was used according to the supplied protocol in all transfections when preparing the stable cell lines.

In the preparations of stabile cell lines, M1 cells were transfected with 1 μg DNA and 1.5 μl Lipofectamine 2000 (Invitrogen, Carlsbad, CA, US) ON, in 500 μl OptiMEM and 1.5 ml DMEM medium. Stable clones with expression of the respective constructs were tested to prove EGFR degradation. Clones with inhibited EGFR degradation were discarded.

### Antibodies and reagents

Anti-Hrs was a gift (Harald Stenmark, NO,^28^). Anti-Eps15 and anti-GFP were obtained from Abcam (Abcam, Cambridge, UK). Anti-Phosphotyrosine clone 4G10 was from Millipore (Merck Millipore, Danvers, MA, US) and anti-EGFP was from Fitzgerald (Fitzgerald, Acton, MA, US). All the secondary antibodies were purchased from GE Healthcare Life Science (Little Chalfont, UK). Both human rec EGF and human rec PDGF BB were obtained from Bachem (Bachem AG, Bubendorf, CH). The EGF-Alexa-647 used in this study was from Life Technologies (Carlsbad, CA, US).

### EGF stimulation and immunoprecipitation

M1 cells were starved for four hours in serum-free medium before stimulation with 100 ng/ml of human rec EGF (Bachem AG, Bubendorf, CH) or 60 ng/ml of human rec PDGF BB (Bachem AG, Bubendorf, CH) for 4, 8, 20 or 40 minutes at 37 °C. Lysis buffer (125 mM Kac, 25 mM Hepes, 25 mM MgAc, 5 mM EGTA, 0,5% NP40, pH 7.2) was supplemented with phosphatase inhibitor cocktail II (P5726-5ML, Sigma-Aldrich, St. Louis, MO, US) and protease arrest (G-Biosciences, St. Louis, MO, US). Immunoprecipitation (IP) was performed according to protocol with Dynabeads Protein G (#63024110, Invitrogen, Carlsbad, CA, US). All the biochemical blots presented in this paper were repeated at least three times.

### Confocal microscopy and live imaging

Cells were grown on chambered cover glass (MatTek Corporation, Ashland, MA, US) and for all the live cell experiments DMEM medium without phenol red and sodium bicarbonate (Invitrogen, Carlsbad, CA, US) were used. Prior to the FRAP experiments the cells were starved for four hours in serum-free medium. The cells were stimulated with 100 ng/ml human rec EGF (Bachem AG, Bubendorf, CH) or 60 ng/ml human rec PDGF BB (Bachem AG, Bubendorf, CH) to stimulate the specific RTKs.

Live imaging experiments were performed on an inverted Olympus iX81 FluoView 1000 confocal microscope (Olympus, Hamburg, DE), equipped with a PlanApo 60x/1.10 oil objective. This microscope has a 4 channel PMT detector unit and a dual SIM scanner specifically designed for high speed FRAP analysis^29^. GFP and YFP were imaged with the multilane Argon laser (457 nm, 488 nm and 515 nm) and mRFP/mCherry with the 559 nm laser. GFP and YFP were bleached with the 405 nm laser, whereas the 559 nm laser was used to bleach mCherry. The endosomes were bleached for 800 milliseconds with maximum laser power.

All photomontages presented were made in Adobe Photoshop CS4 and Adobe InDesign CS4.

### Colocalization analysis

The colocalization analysis was performed with ImageJ (http://rsbweb.nih.gov/ij/). For the analysis of single images and the analysis of Eps15-GFP and Hrs-mRFP colocalization the JACoP plugin (http://rsbweb.nih.gov/ij/plugins/track/jacop.html) was used to calculate the “overlap coefficient”. Analysis of EGF-Alexa-647 colocalization with Eps15-GFP or Hrs-mRFP through time was an object based analysis and calculating the Pearsons´s coefficient was performed in GraphPad Prism 4 (http://www.graphpad.com/scientific-software/prism/).

### The endosomal fluorescence intensity analysis

To calculate the endosomal fluorescence intensity, 10 individual endosomes from 10 independent experiments were measured at the specific time points, before and after stimulation with 100 ng/ml human rec EGF (Bachem AG, Bubendorf, CH) or 60 ng/ml human rec PDGF BB (Bachem AG, Bubendorf, CH). The fluorescence endosomal intensity and the total cellular intensity were measured with the Series Analysis profile in the FV10-ASW4.2 software from Olympus (Olympus, Hamburg, DE). To correct for the general bleaching the total cellular fluorescence intensity was subtracted from the measured endosomal fluorescence intensity. The endosomal fluorescence intensity at time point zero was set to 1 and calculated for each time point for each individual endosome was normalized according to this value.

### FRAP analysis

Obtained data from FRAP was normalized and corrected for bleaching^30^ and fitted by nonlinear regression to a function that assumes a single diffusion coefficient^31^;

1 $$\,F(t)=(F(0)+(F(\infty )({\rm{t}}/{\rm{t}}1/2)))/(1+({\rm{t}}/{\rm{t}}1/2))$$

The values for *F(0*), *F(∞)* and *T*_*1/2*_ were calculated using GraphPad Prism 6 (http://www.graphpad.com/scientific-software/prism/) and the immobile fractions (IF) were calculated as described in Lippincott-Schwartz *et al*.^32^.

### The total mobile fraction (tMF)

The fraction of molecules that is slowly exchanged between bleached and non-bleached regions is called the immobile fraction (IF). The mobile fraction, defines the molecules freely exchanged between the bleached and non-bleached region presented by: MF = (1-IF). If the two fractions at the fluorescent endosomal membrane proteins are altered after RTK activation we need independent measurements monitoring the total endosomal fluorescence intensity, tEFI. The tEFI for each endosome is normalized to 1 before stimulation. The corresponding total immobile fraction (tIF) and total mobile fractions (tMF) may then be calculated as followed:

2 $${\rm{tIF}}={\rm{IF}}\times {\rm{tEFI}}$$

3 $${\rm{tMF}}={\rm{tEFI}}-{\rm{tIF}}$$

In the experiments in this study the tEFI values and the immobile fraction (IF) and mobile fraction (MF) represents the average of at least 10 endosomes for each time point.

### Degradation assay

Cells stably expressing Hrs-YFP, HrsY329/334F-YFP, Eps15-GFP or Eps15Y850F-GFP were incubated with or without EGF (60 ng/ml)/PDGF (60 ng/ml) and cyclohexamid (10 ug/ml) for 0 h, 2 h or 4 h at 37 °C. The receptor degradation assays for each of these four cell lines was repeated 3 times and the quantification was performed with ImageQuant (http://www.imsupport.com/).

## Results

### Internalized EGF-Alexa-647 localizes to Eps15-GFP/Hrs-mRFP positive endosomes

Ligand bound EGF-R is internalized and is degraded in MVBs^33,34^. Important regulators for the trafficking and degradation of EGF-R are Hrs and Eps15^12,33^.

**Figure 1:**
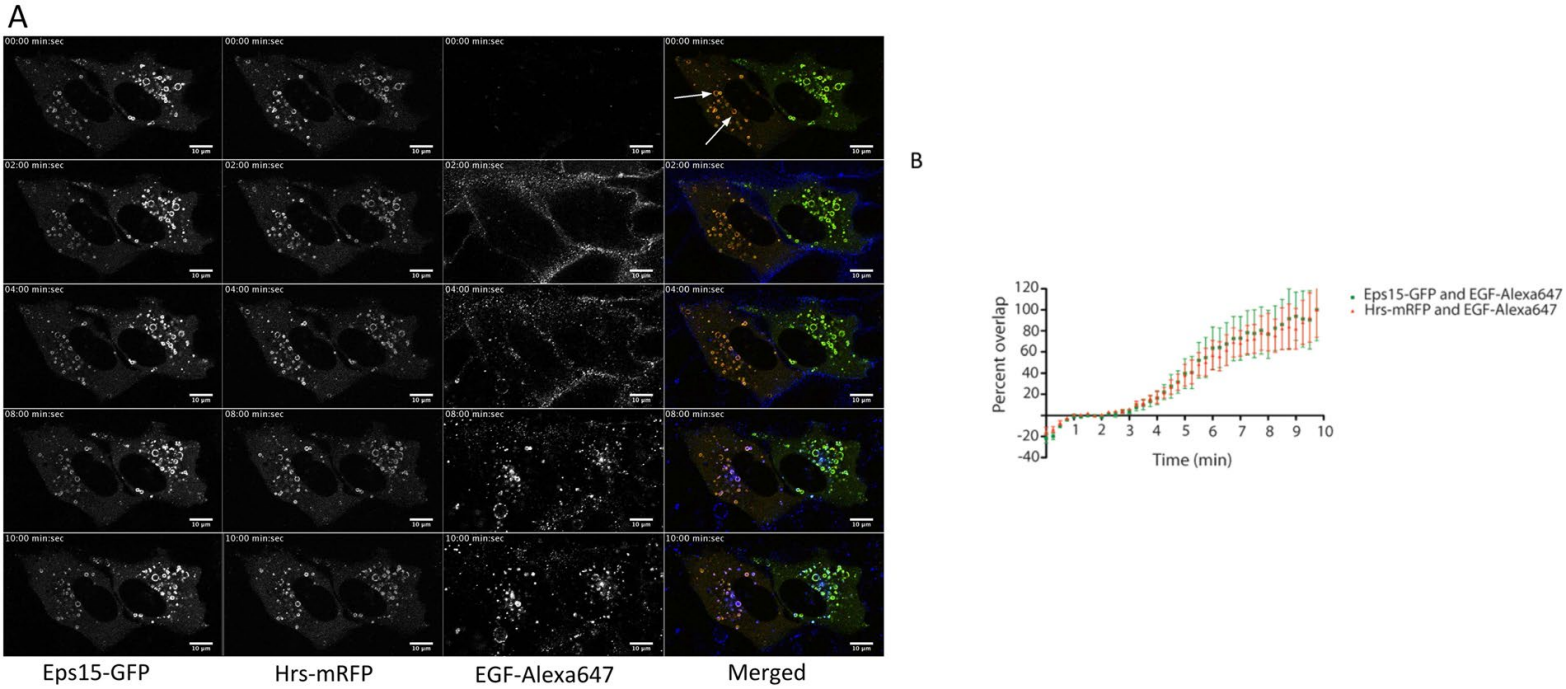
EGF-Alexa-647 localizes to endosomes positive of Eps15-GFP and Hrs-mRFP. (**A**) A time series montage with M1 cells stably expressing Ii-pMep4 and co-transfected with Eps15-GFP and Hrs-mRFP, EGF-Alexa-647 were added during imaging. Eps15-GFP and Hrs-mRFP localize to the same enlarged endosomes prior to EGF stimulation (white arrows). EGF-Alexa-647 are internalization and localization to enlarged Eps15-GFP/Hrs-mRFP positive endosomes. (**B**) The graph signifies an object based colocalization analysis through time. The graph shows the overlap between Eps15-GFP/EGF-Alexa-647 (green colour) and Hrs-mRFP/EGF-Alexa-647 (red colour) in overlapping regions on enlarged endosomes. The graph shows the results from 5 independent experiments (mean values ± s.d).

M1 cells stably transfected with Ii were co-transfected with either Eps15-GFP or Hrs-mRFP. Ii induced overnight, resulted in the characteristic enlarged endosomes^16–20^, which were found to be positive for Eps15-GFP and Hrs-mRFP (Fig. 1A). Both of the fluorescent proteins were detected in cytosol and on enlarged endosomes. Membrane associated Eps15-GFP and Hrs-mRFP were found in overlapping regions at on the endosomal membrane (Fig. 1A, white arrows, Supplementary Movie 1). Analysis of the overlapping Eps15-GFP and Hrs-mRFP domains showed an overlap-coefficient of 89% ± 4% prior to EGF stimulation.

To further follow the route of EGF ligand through the pathway of enlarged endocytic organelles, EGF-Alexa-647 was added to the cells while imaging. Labelled EGF was first detected at the PM and was sequentially internalized within two minutes after ligand addition (Fig. 1A,B). Importantly, the transport of EGF-Alexa-647 from the PM to the Eps15-GFP/Hrs-mRFP positive endosomes was fast and we could detect endosomal localization of EGF-Alexa-647 3-4 minutes after addition. We could observe EGF-Alexa-647 specifically localizing to endosomes positive for both Eps15-GFP and Hrs-mRFP (Fig. 1A,B). Following the endosomal localization through time we could observe an increasing recruitment of EGF-Alexa-647 to the Eps15-GFP and Hrs-YFP positive endosomes. Analysing the correlation between the two colocalization-graphs (Fig. 1B), shows that the Pearson coefficients (see methods) were almost identical, r = 0.98 for Eps15-GFP and EGF-Alexa-647 and r = 0.99 for Hrs-YFP and EGF-Alexa-647. This indicates a specific trafficking of the ligand bound EGF-R to endosomal vesicles positive for Eps15-GFP and Hrs-mRFP.

### Ligand stimulation induces a transient change in the endosomal fluorescence intensity of Eps15-GFP and Hrs-YFP

**Figure 2:**
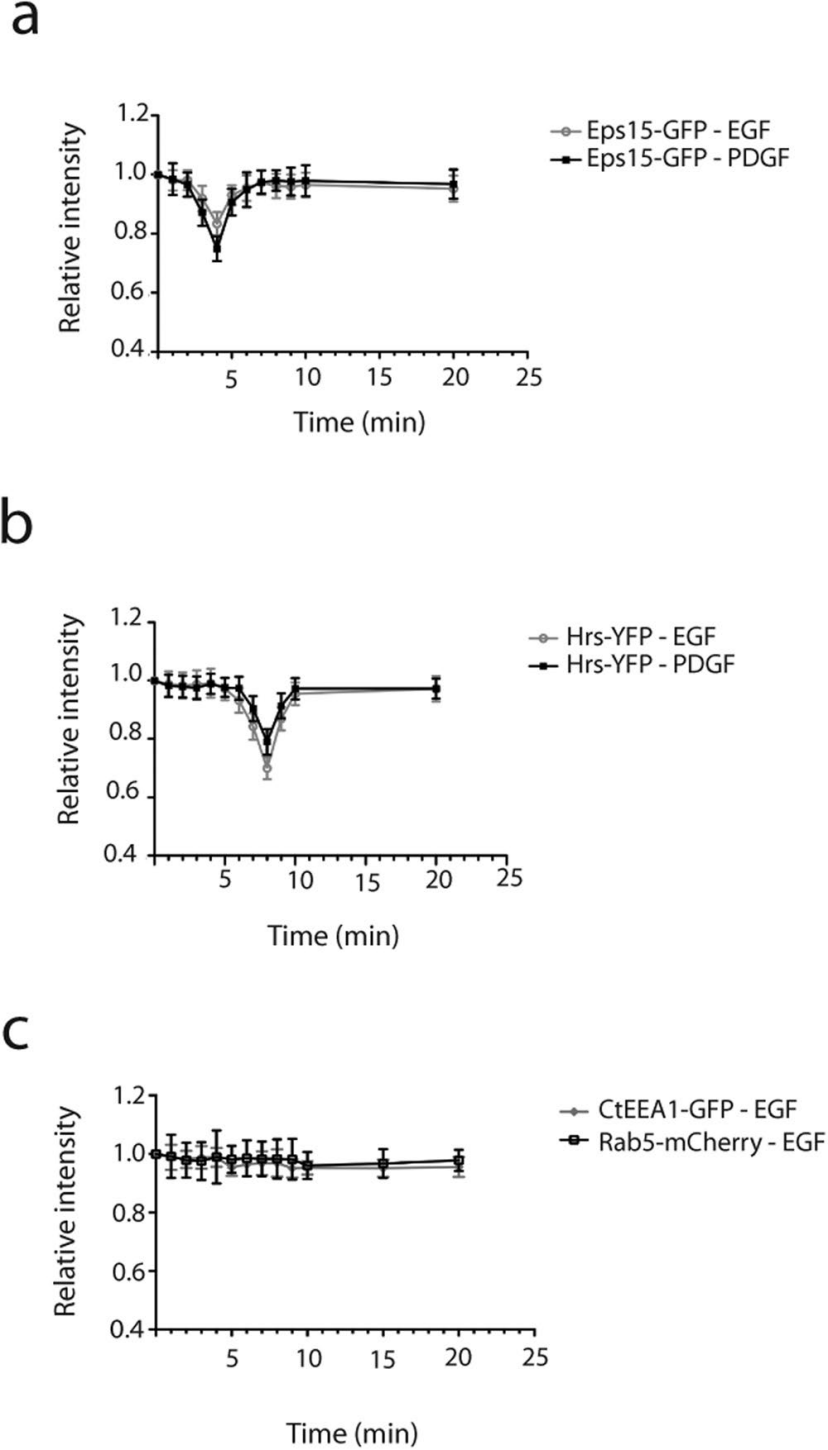
EGF and PDGF stimulation induce a transient drop in fluorescence of Eps15-GFP or Hrs-YFP on enlarged endosomes. M1 cells stably expressing Ii and Eps15-GFP or Hrs-YFP were stimulated with EGF/PDGF. Data represents the mean ± s.d of 10 independent experiments. (**a**) The graph shows the fluorescence intensity of endosomal Eps15-GFP after ligand stimulation. P value 0.0003 (***) after EGF and < 0.0001 (****) after PDGF stimulation. (**b**) The graph shows the fluorescence intensity of endosomal Hrs-YFP after ligand stimulation. The P value < 0.0001 (****) both after EGF- and PDGF stimulation. (**c**) Control experiments of M1 cells stably expressing CtEEA1-GFP or Rab5-mCherry after EGF stimulation.

Following the trafficking of labelled EGF through Hrs-mRFP and Eps15-GFP positive endosomes (Fig. 1) we could measure a transient drop in the fluorescence intensity of both fusion proteins after EGF-R activation (Fig. 2).

By tracking 10 single endosomes positive for Eps15-GFP after EGF stimulation from 10 independent experiments, we could with the Series Analysis profile in the Olympus software measure a 17% ± 12.4% transient decrease in the fluorescence intensity 4 minutes (Fig. 2a). Subsequently, eight minutes after stimulation the endosomal fluorescence intensity for Eps15-GFP was restored (Fig. 2a).

Similarly, we investigated the fluorescence intensity of Hrs-YFP on the endosomal membrane upon EGF stimulation in 10 independent experiments. Reminiscent of the results with Eps15-GFP we could calculate a 30% ± 11,9% decrease in the intensity of Hrs-YFP on single endosomes eight minutes after EGF stimulation (Fig. 2b). Furthermore, 10 minutes after EGF treatment the endosomal intensity of Hrs-YFP was restored to the initial intensity prior to stimulation.

Eps15 has also been identified as a component in Platelet-derived growth factor receptor (PDGF-R) signalling, and is phosphorylated in response to activation of the receptor^35,36^. To test whether the alteration in endosomal fluorescence intensity is a specific mechanism in EGF-R activation or a more general effect after activation of RTKs we performed identical experiments with the PDGF-R stimulated by PDGF. Interestingly, also activation of PDGF-R induced a significant transient drop of fluorescence intensity of both Hrs-YFP and Eps15-GFP, consistent with the time point for EGF-R (Fig. 2a,b).

To establish the specificity of the above mentioned transient fluorescence drop for Eps15-GFP and Hrs-YFP, we analysed if other proteins that is recruited to early endosomes could be affected by EGF stimulation. M1 cells were transfected with two well-known early endosomal markers, Rab5mCherry and CtEEA1-GFP (residues 1257–1411 of EEA1, see material and methods,^26^). After EGF stimulation the endosomal fluorescence intensity of these proteins remained constant (Fig. 2c). These data show that a transient drop of the proteins recruited to early endosomes are not a general effect. Together the results above indicate that an activation of RTKs specifically regulate the level of Hrs and Eps15 bound to the endosomal membrane.

### EGF-R activation change the membrane to cytosol binding dynamics of Eps15-GFP and Hrs-YFP

In stably transfected cells, Eps15-GFP and Hrs-YFP were localized to endosomal membranes and in the cytosol (Fig. 1A). The EGF-induced change in Eps15-GFP and Hrs-YFP fluorescence intensity on the endosomal membrane most likely due to a change in the endosomal binding kinetics of the two proteins.

**Figure 3:**
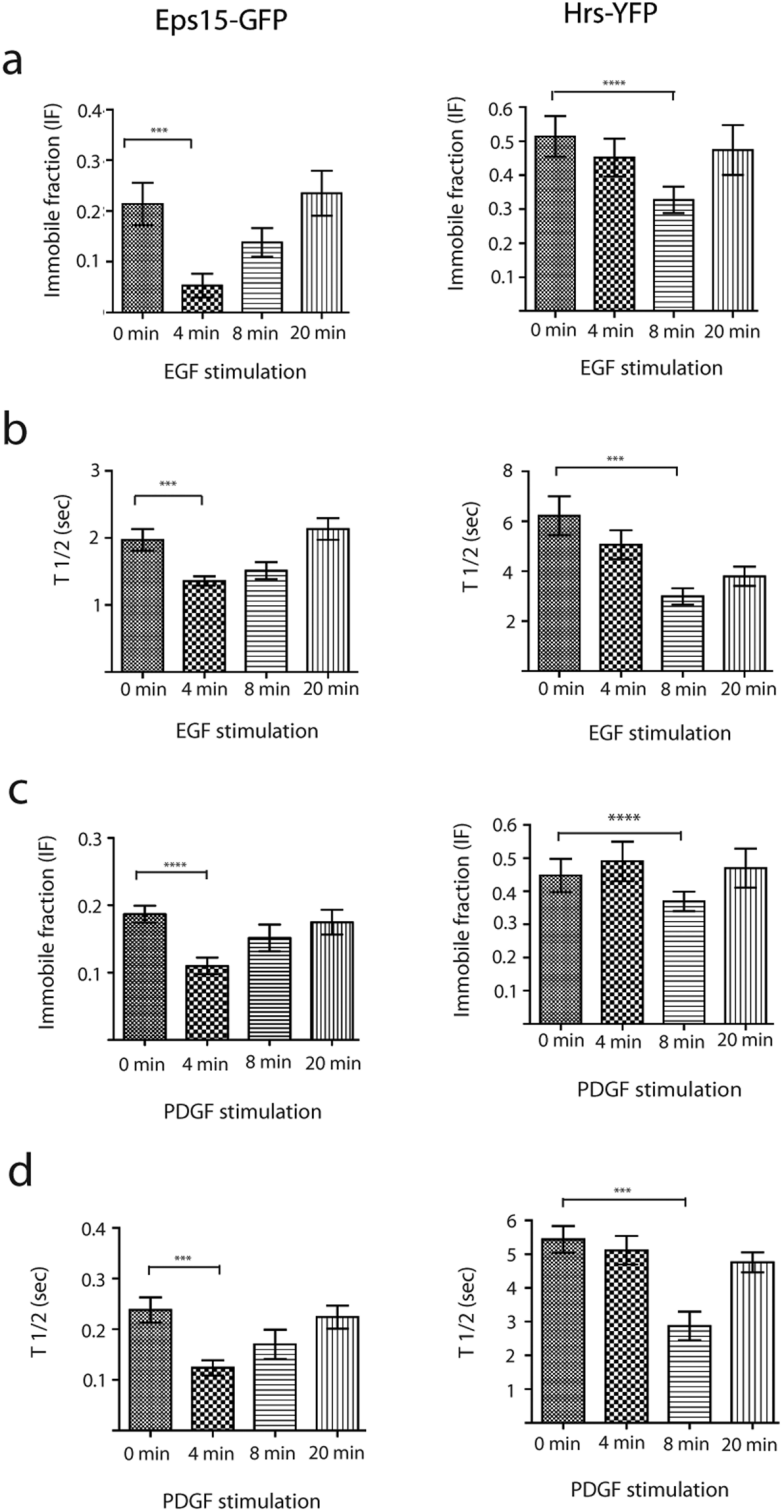
EGF and PDGF stimulation induce a transient change in the binding kinetics of Eps15-GFP and Hrs-YFP. M1 cells were serum starved for 4 hours before the FRAP experiments. Single enlarged endosomes positive for Eps15-GFP or Hrs-YFP were bleached at specific time points (0, 4, 8 and 20 minutes) after EGF/PDGF stimulation. All data represents in this figure is the mean value of 10 independent experiments ± s.d. (**a**) The graph shows the immobile fraction (IF) for Eps15-GFP and Hrs-YFP at specific time points after EGF stimulation. P value < 0.0003 (***) for Eps15-GFP and P < 0.0001 (****) for Hrs-YFP. (**b)** Graph showing the T_1/2_ recovery for Eps15-GFP and Hrs-YFP at similar time points as in (A). P value < 0.0006 (***) for Eps15-GFP and P value 0.0009 (***) for Hrs-YFP. (**c**) The graph shows the IF for Eps15-GFP and Hrs-YFP at specific time points after PDGF stimulation. P < 0.0001 (****) for Eps15 and P < 0.0001 (****) for Hrs-YFP. (**d**) Graph showing the T_1/2_ recovery for Eps15-GFP and Hrs-YFP at specific time points after PDGF stimulation. P value < 0.0001 (***) for Eps15-GFP and P value 0.0001 (***) for Hrs-YFP.

To investigate this further, we bleached single enlarged endosomes positive for Eps15-GFP or Hrs-YFP and measured the recovery of the fluorescent proteins. Based on these Fluorescence Recovery After Photobleaching (FRAP) experiments we calculated the endosomal T_1/2_ recovery and the immobile fraction at different time points after EGF stimulation (see materials and methods,^37^). We could measure a significant decrease of the immobile fraction for Eps15-GFP and Hrs-YFP and also a reduction in the T_1/2_ (Fig. 3a,b, Supplementary Table 1). Intriguingly, the transient change in the binding kinetics occurred at the same time points as the endosomal fluorescence intensity of Eps15-GFP or Hrs-YFP decreased (Fig. 2a,b). We could specifically measure a 76.3% drop in immobile fraction after four minutes for Eps15-GFP and a 39.6% drop after eight minutes for Hrs-YFP (Fig. 3a, Supplementary Table 1). Additionally, EGF stimulation induced a 31% decrease in T_1/2_ recovery for Eps15-GFP and 52% decrease for Hrs-YFP (Fig. 3b, Supplementary Table 1).

We further tested whether PDGF-R activation could induce a similar change in the cytosol to endosome binding kinetics for Eps15-GFP and Hrs-YFP. Reminiscent of EGF-R activation, a transient change in the binding kinetics of Eps15-GFP and Hrs-YFP four and eight minutes after PDGF stimulation was measured (Fig. 3c,d, Supplementary Table 2).

Previous experiments with CtEEA1-GFP and Rab5mCherry did not show any change in the endosomal membrane fluorescence intensity after EGF stimulation. By performing FRAP experiments on single enlarged endosomes positive for CtEEA1-GFP or Rab5mCherry, we found that both the immobile fraction and T_1/2_ recovery remained constant after EGF stimulation (Supplementary Table 1). This shows that the endosomal binding kinetics for CtEEA-GFP and Rab5mCherry is unaffected by EGF stimulation in contrast to Eps15-GFP and Hrs-YFP.

**Figure 4:**
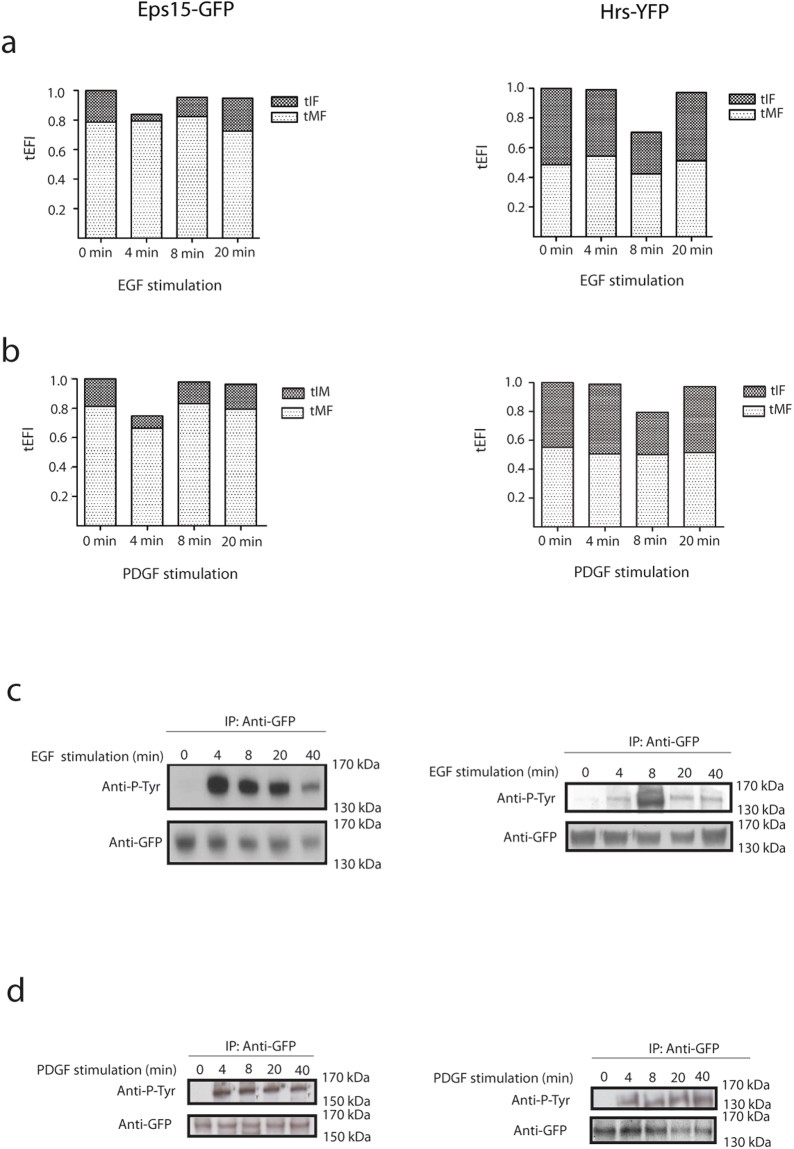
Change in the binding kinetics is induced by phosphorylation. (**a**) This figure shows the total Endosomal Fluorescence Intensity (tEFI) for Eps15-GFP and Hrs-YFP after EGF stimulation divided into their significant fractions, tIF (dark grey) and tMF (light grey). (**b**) This figure shows the total Endosomal Fluorescence Intensity (tEFI) for Eps15-GFP and Hrs-YFP after PDGF stimulation divided into their significant fractions, tIF (dark grey) and tMF (light grey). (**c**) Analysis of the Eps15-GFP and Hrs-YFP phosphorylation level after EGF stimulation. Eps15-GFP and Hrs-YFP were IP with anti-GFP and the phosphorylation level was detected with an anti-phosphotyrosine antibody. The total protein levels of Eps15-GFP and Hrs-YFP after IP were detected with an anti-GFP antibody. The western blot was repeated 3 times. (**d**) Analysis of the Eps15-GFP and Hrs-YFP phosphorylation level after PDGF stimulation. Eps15-GFP and Hrs-YFP were IP with anti-GFP and the phosphorylation level was detected with an anti-phosphotyrosine antibody. The total protein levels of Eps15-GFP and Hrs-YFP after IP were detected with an anti-GFP antibody. The western blot was repeated 3 times.

We have above described an essential EGF induced change in the T_1/2_ recovery for the mobile fraction and the immobile fraction for Eps15-GFP and Hrs-YFP. However, we were also interested in characterizing how the individual fractions were altered by EGF stimulation. To analyse the dynamics of the two fractions on the unbleached endosomes we combined the calculations of the total endosomal fluorescence intensity (tEFI) with measured immobile and mobile fractions (Fig. 3a) (see materials and methods). From these calculations, we could show that the total mobile fraction (tMF) remained relatively stable, while a major decrease in the total immobile fraction (tIF) of both Eps15-GFP and Hrs-YFP was observed (Fig. 4a). Similar calculations were done for the PDGF-R with the same results (Fig. 4b). This change in the immobile fraction might explain the previously observed relocation of Hrs from endosomes to cytosol^21,22^ and we could further show similar rearrangement for Eps15.

Together these changes in endosomal fluorescence intensity and membrane to cytosol cycling indicate an altered mechanism in binding dynamics of Eps15-GFP and Hrs-YFP. This time dependent change in membrane fraction proved to be a redistribution of the immobile fraction.

### Ligand activation induce a transient increase in phosphorylation of Eps15-GFP and Hrs-YFP

It has previously been described that endogenous Eps15 and Hrs become phosphorylated upon EGF-R activation^38,39^. We wanted to further, biochemically analyse whether there could be an association between the change in binding kinetics and the phosphorylation of the Eps15-GFP and Hrs-YFP. When probing with an antibody against phosphorylated tyrosine residues, we could measure an increased level of phosphorylation for Eps15-GFP after four minutes and for Hrs-YFP after eight minutes, subsequently of EGF stimulation (Fig. 4c). The increased level of phosphorylation corresponded with the time same points as the transient changes in membrane binding kinetics could be measured. Similar experiments were performed with PDGF stimulation to further verify the coexistence of increased phosphorylation and changed binding dynamics of Eps15-GFP and Hrs-YFP. When stimulating the PDGF-R we could measure a simultaneous phosphorylation after four minutes for Eps15-GFP and Hrs-YFP. Differently from the EGF stimulation we did not observe any consecutive increased phosphorylation with four minutes difference, after PDGF-R activation (Fig. 4d). This suggests that different time dependent phosphorylation characteristics of Eps15-GFP and Hrs-YFP induce a similar regulatory effect on their binding kinetics.

### Ligand induced phosphorylation of Eps15-GFP and Hrs-YFP regulates the endosome to cytosol kinetics

The preceding experiments indicated a phosphorylation–mediated mechanism regulating the binding kinetics of Eps15-GFP and Hrs-YFP. To test whether phosphorylation is a prerequisite for the change in binding kinetics we point-mutated the known phosphorylation site Y850 in Eps15-GFP and in Hrs-YFP, the two adjacent phosphorylation sites Y329 and Y334^7,15^.

**Figure 5:**
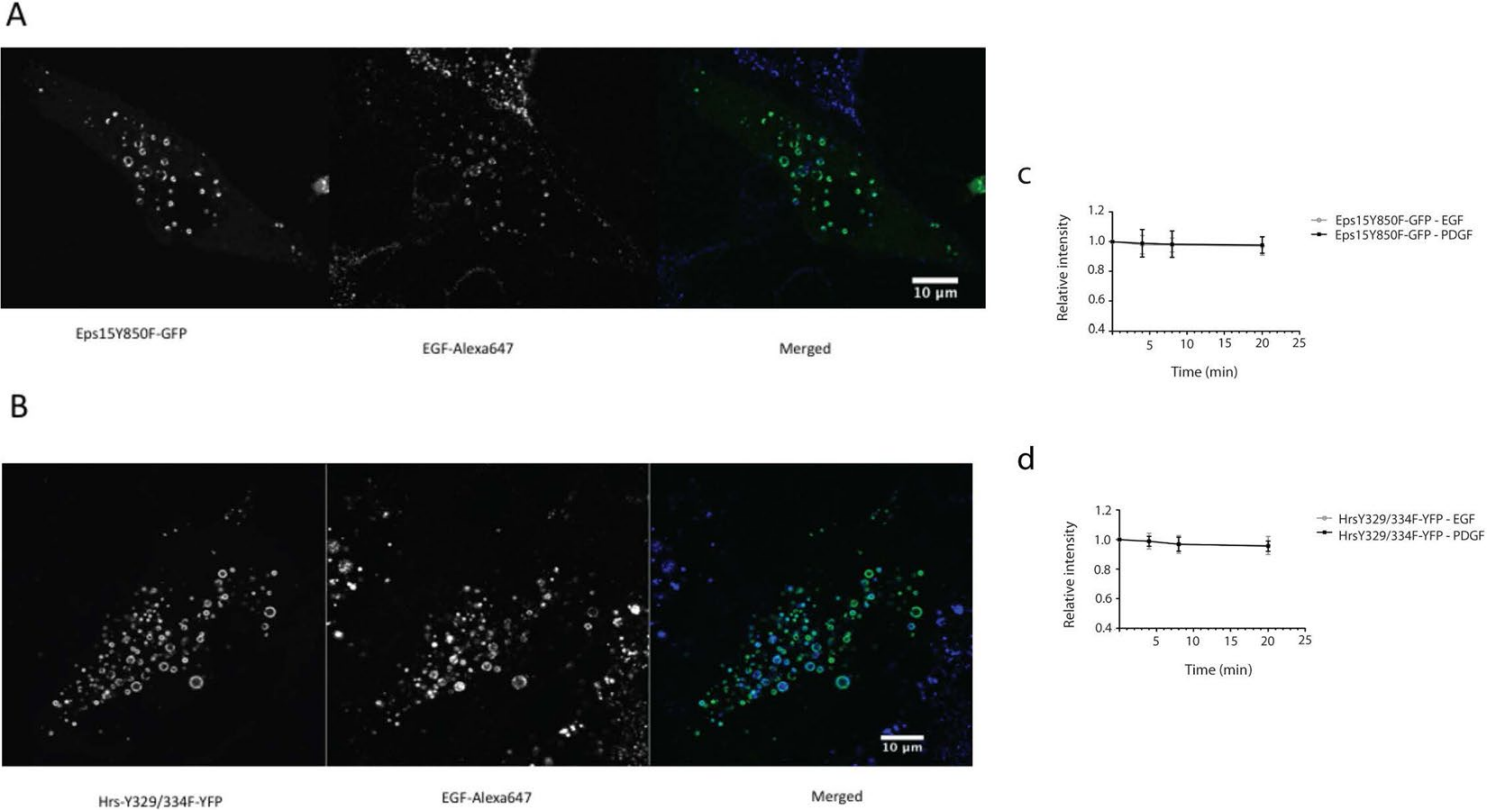
Endosomally located phosphorylation deficient Eps15-GFP and Hrs-YFP are unaffected by ligand stimulation. (**A**) montage showing EGF-Alexa-647 internalization and localization to enlarged Eps15Y850F-GFP endosomes. (**B**) A montage showing EGF-Alexa-647 internalization and localization to enlarged HrsY329/334F-YFP endosomes. (**c**) This graph shows the endosomal fluorescence intensity of the Eps15 tyrosine mutant (Eps15Y850F-GFP) after stimulation with unlabelled EGF/PDGF. Data represents the mean value of 10 independent experiments ± s.d. (**d**) This graph shows the endosomal fluorescence intensity of the of the Hrs-YFP tyrosine mutant (HrsY329, 334F-YFP) after stimulation with unlabelled EGF/PDGF. Data represents the mean value of 10 independent experiments ± s.d.

M1 cells stably transfected with Ii were co-transfected with either Eps15Y850F-GFP or HrsY329/334F-YFP. Ii was induced overnight, resulting in characteristic enlarged endosomes and fluorescently labelled EGF was added to the cells. Concomitant with cells expressing the wt fusion constructs (Fig. 1) we could observe endosomal colocalization of internalized EGF-Alexa-647 (Fig. 5A,B), indicative of a well functioning internalization and trafficking of the receptors to the endosomes in the mutant transfected cells.

**Figure 6:**
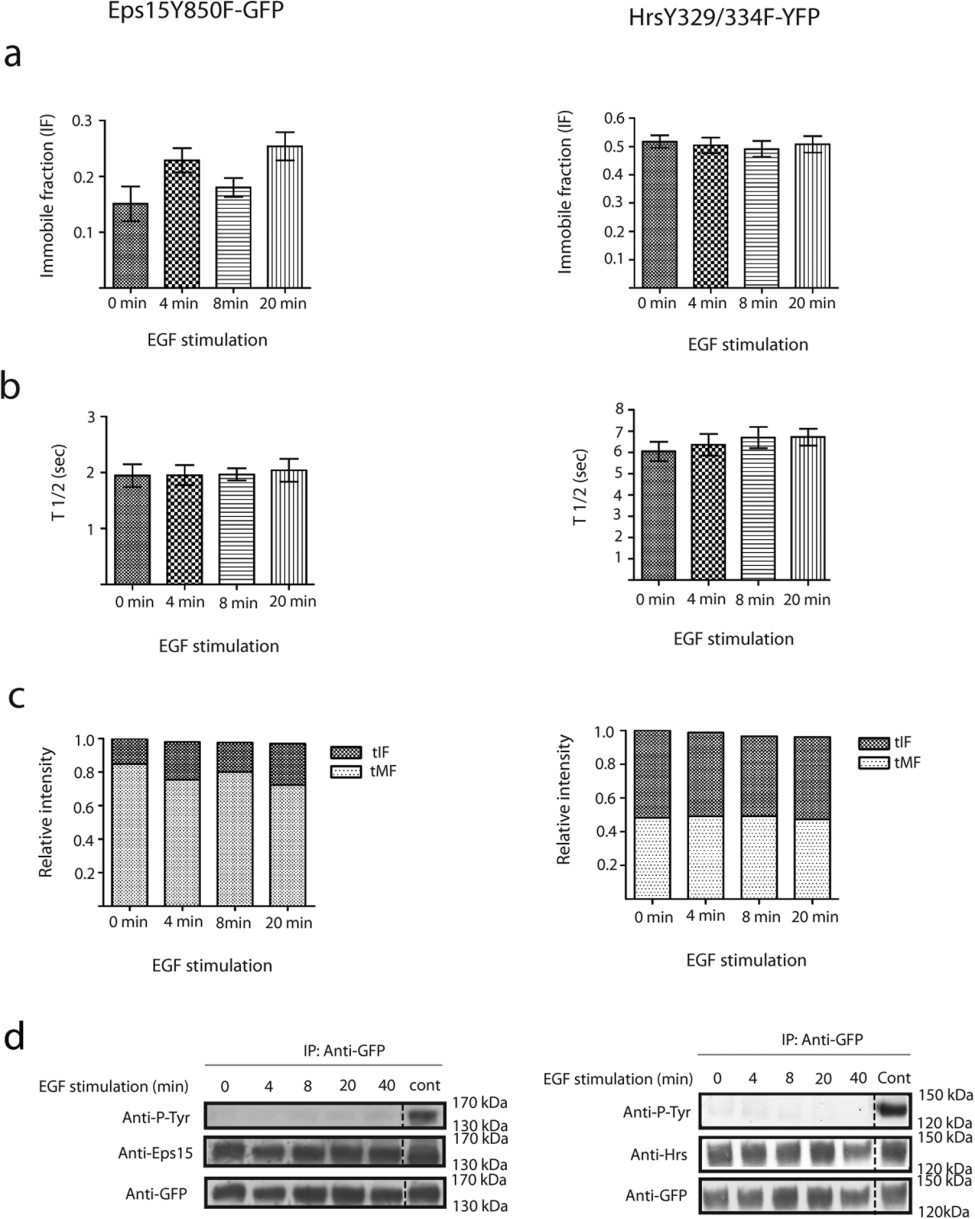
Receptor stimulation does not change the endosomal binding kinetics of tyrosine mutated Eps15-GFP and Hrs-YFP. M1 cells were serum starved for 4 hours before the FRAP experiments. Single enlarged endosomes positive for Eps15Y850F-GFP or HrsY329/334F-YFP were bleached at specific time points (0, 4, 8 and 20 minutes) after EGF stimulation. (**a**) These graphs show the IF for Eps15Y850F-GFP and HrsY329/334F-YFP at specific time points after EGF stimulation. Data represents the mean of 10 independent experiments ± s.d. (**b**) Graph showing the T_1/2_ recovery for Eps15Y850F-GFP and HrsY329/334F-YFP. Data represents the mean of 10 independent experiments ± s.d. (**c**) This figure shows the total Endosomal Fluorescence Intensity (tEFI) for Eps15Y850F-GFP/HrsY329/334F-YFP, divided into their significant fractions, tIF (dark grey) and tMF (light grey). (**d**) Analysis of the tyrosine mutated Eps15 and Hrs after stimulation with EGF. Eps15Y850F-GFP or HrsY329/334F-YFP was IP with anti-GFP and the phosphorylation level was detected with an anti-phosphotyrosine antibody. To confirm that Eps15Y850F-GFP or HrsY329/334F-YFP were present on the membrane antibodies against Eps15/Hrs were used. The total levels of tyrosine mutants after IP were detected with an anti-GFP antibody. The positive controls in these experiments are Eps15-GFP wt (stimulated with EGF for 4 minutes) or Hrs-YFP wt (stimulated with EGF for 8 minutes). The western blot experiments were repeated 3 times. The dashed line indicates the removed ladder lane.

Similar measurements as we did for the wt (Fig. 2a,b), were done to investigate the fluorescence intensity on endosomes positive for the mutant constructs (Fig. 5c,d). In contrast to the cells expressing the wt constructs (Fig. 2a,b) we could not measure any ligand-induced change in the endosomal fluorescence intensity. FRAP analysis could further show no time dependent change in the endosomal immobile fraction or the T_1/2_ recovery in EGF nor PDGF stimulated cells expressing the mutant fusion proteins (Fig. 6a,b Supplementary Tables 1 and 2). The tEFI were calculated for both mutants after EGF/PDGF stimulation (see materials and methods). In contrast to cells expressing the wt construct the tMF and tIF remain relatively stabile for both mutants (Fig. 6c).

Our previous analysis showed us that Eps15-GFP and Hrs-YFP became phosphorylated upon EGF-R activation (Fig. 4c). Similar biochemical analyses were done for the two mutated proteins, Eps15Y850F-GFP and HrsY329F/Y334F-YFP. In contrast to the wt construct we could here show that we were no detectable phosphorylation for the two mutants (Fig. 6d).

This indicated that the EGF-R cannot regulate the endosomal binding dynamic of tyrosine mutated Eps15-GFP or Hrs-YFP and that phosphorylation of Eps15-GFP and Hrs-YFP is crucial for regulating their endosomal binding kinetics.

### Phosphorylation of Eps15-GFP and Hrs-YFP regulates the EGF-R and PDGF-R degradation

**Figure 7:**
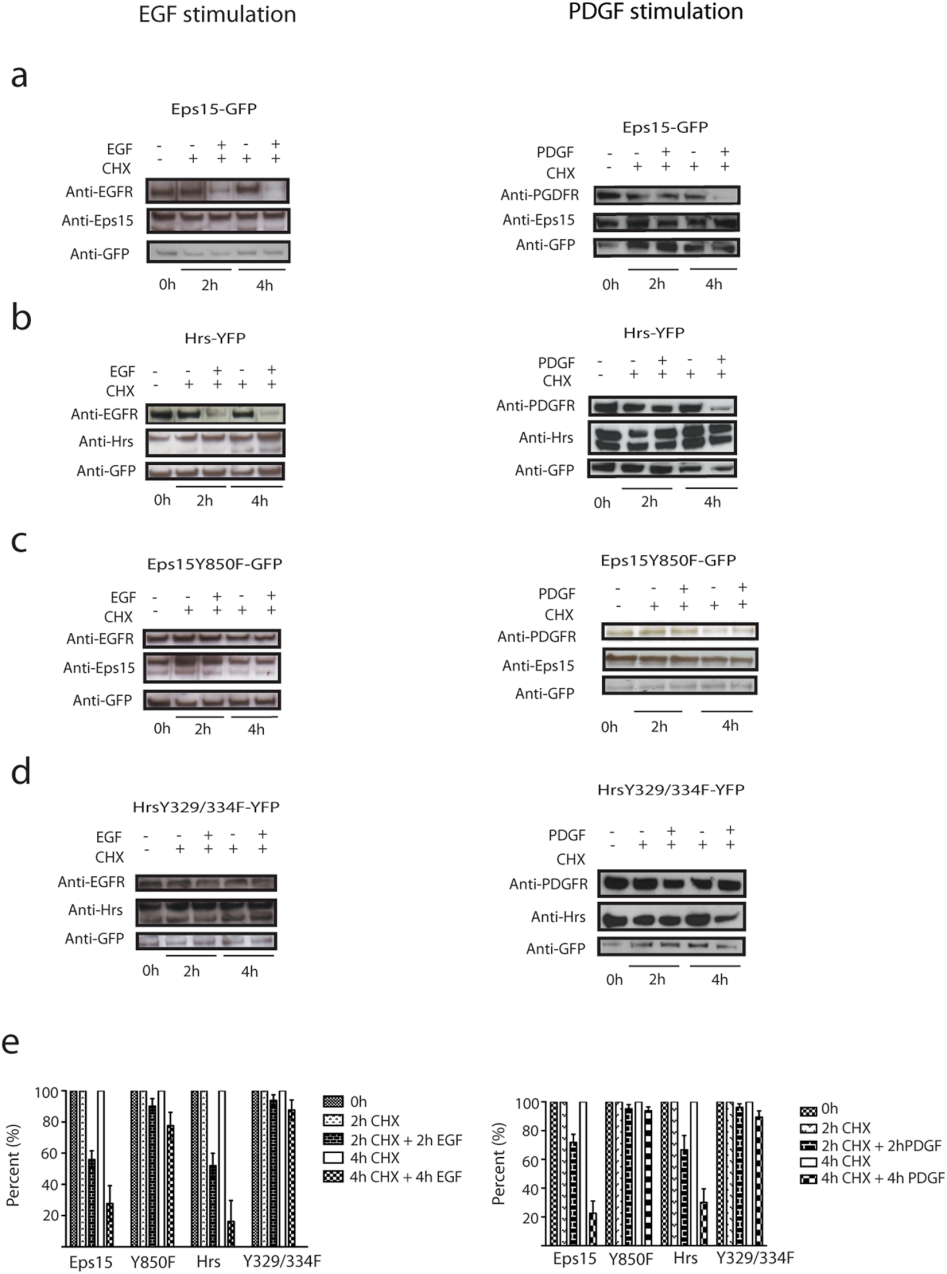
The mutants Eps15Y850F-GFP and HrsY329/334F-YFP reduce the EGF-R and PDGF-R degradation. The figure shows an EGF-R degradation assay on M1 cells stably expressing Ii-pMep4, co-transfected with either (**a**) Eps15-GFP, (**b**) Hrs-YFP, (**c**) Eps15Y850F-GFP or (**d**) HrsY329/334F-YFP. The cells were treated with cycloheximide (CHX) and stimulated with unlabelled EGF or PDGF BB. The amount of EGF-R was analysed with an antibody against EGF-R, and the total amount of proteins present on the membrane was detected with antibodies against Eps15/Hrs/GFP. Quantification of EGF-R (**e**) and PGDF-R (**f**) degradation assay are based on three individual experiments.

Impaired endosomal progression will inhibit receptor degradation. When analysing the degradation of the EGF-R and PDGF-R, in our stably transfected cells, we observed a major degradation after two and four hours (Fig. 7a,b,e). However, in the cells stably transfected with the phosphorylation deficient mutants we could measure an inhibition of receptor degradation for both receptors (Fig. 7c–e). These results show that phosphorylation of Eps15 and Hrs is essential for efficient EGF-R and PDGF-R degradation, supporting the above data that phosphorylation of these molecules influence progression and maturation in the endosomal pathway.

## Discussion

Specific regulation of the RTKs is mediated through signalling and endocytic trafficking^41^. Protein modifications such as phosphorylation or ubiquitinylation have the potential to modify this complex process. By stimulating the RTKs, EGF-R and PDGF-R, we have been able to study their downstream effects on endosomal membrane binding kinetics of Eps15-GFP and Hrs-YFP.

To better study single endosomal vesicles, we enlarged their size by expressing the Ii in the human cell line M1^18–20,37,42^. This is a well-established method where Ii expression enlarges the size of the endosomes without interfering with the important Rab5 to Rab7 transition^17,40,43^. Importantly, previous studies could show that the Ii induced enlargement did not change the membrane binding dynamics of the endosomal tethering-molecule EEA1^37^. We could furthermore confirm a similar EGF-R induced phosphorylation pattern for Eps15 and Hrs in M1 cells expressing Ii as in untransfected cells^7,9,15,38,39,44^.

In M1 cells transfected with Eps15-GFP and Hrs-mRFP we observed that the two proteins are present on the same Ii enlarged endosomes prior to ligand stimulation (Fig. 1A). When stimulating the cells with labelled EGF we could follow the internalization and transport of EGF from the PM through the endosomal pathway. EGF was specifically transported from the PM to the endosomes positive for Eps15-GFP and Hrs-mRFP. Mutual endosomal localization of EGF-R cargo with Eps15-GFP and Hrs-mRFP indicates a shared role for endosomal Eps15 and Hrs, either for recruitment of the receptor to endosomes or for subsequent downregulation^12,33^.

EGF-Alexa-647 was transported from the PM to the endosomes positive for Eps15-GFP/Hrs-mRFP in just over two minutes (Fig. 1A,B). When EGF-Alexa-647 was detected on enlarged endosomes, we could concomitantly measure a significant transient decrease in the fluorescent signal of Eps15-GFP and Hrs-YFP (Fig. 2a,b). This shows that ligand bound EGF-R, present on the endosomal membrane, can induce a local change in the level of Eps15 and Hrs and that ligand bound RTKs may through endosomal signalling, locally regulate the level of Eps15 and Hrs on endosomes.

The binding kinetics of Eps15-GFP and Hrs-YFP measured by FRAP show significant variations both in the T_1/2_ for the recovery and the immobile fraction after ligand stimulation (Fig. 3a,b). Interestingly, the EGF induced changes in the binding kinetics of Eps15-GFP and Hrs-YFP were detected at the same time points as the decrease in endosomal fluorescence. This demonstrates that an activated EGF-R can specifically regulate the endosomal level of Eps15 and Hrs by controlling the endosome to cytosol binding kinetics. This endorses an endosomal signalling effect of a ligand bound RTK, locally administered on the endosomes.

From our experiments, we could measure a differential EGF-R regulation of the tMF and the tIF of both Eps15-GFP and Hrs-YFP on the endosomes. Activated EGF-R could free more of the immobile fraction of Eps15-GFP after 4 minutes compared to the immobile fraction of Hrs-YFP after 8 minutes. We have in an earlier study proposed that the immobile fraction of the tethering molecule EEA1 bound to the endosomes is the regulatory fraction of the binding kinetics^37^. Given this, it is interesting to observe that for Eps15 and Hrs the intensity drop after EGF activation can be accounted for entirely by a reduced immobile fraction (Fig. 3a). Specifically analysing the tIF of Eps15-GFP we can measure a 79% reduction after ligand stimulation. This suggests a unique mechanism where a change in the immobile fraction shifts the phosphorylated molecule from the endosomal membrane to the cytosol. This demonstrates for the first time that the immobile fraction is not stationary, but an adjustable fraction specifically regulated by the RTK receptors.

The Eps15 and Hrs regulation is also time dependent as discussed above and occurred 4 and 8 minutes after addition of ligand to the cells similar to the earlier studies of sequential phosphorylation^7,15^. When mutating the main phosphorylation sites in Eps15-GFP and Hrs-YFP, the EGF stimulation did not induce any change in the endosomal membrane to cytosol binding of the mutated proteins (Fig. 6a,b).

However, both mutants inhibited degradation of the EGF-R and PDGF-R (Fig. 7). This is in line with earlier data in HEK293 cells showing that a similar Hrs phosphorylation mutant inhibited EGF-R degradation and stimulation of phosphorylation increased degradation^21^.

We have here shown that RTK induced phosphorylation of Eps15 and Hrs reduced endosomal binding of these molecules and the phoshorylation deficient Eps15 and Hrs inhibit EGF-R and PDGF-R degradation. The ESCRT complex mediates degradation by controlling the entry into the MVBs^3^. Our data support previous work showing that non-phosphorylated Hrs blocks the entry into the ESCRT mediated EGF-R degradation pathway^21^. Previous data published have shown that the majority of phosphorylated Hrs is present in the cytosol and that phosphorylation occurs on the endosome^15,22^. Our data shows that Eps15 and Hrs are present on the endosomal membranes and in cytosol before ligand activation. Receptor stimulation induces endosomal sequential phosphorylation of Eps15 and Hrs on the endosomes as an incentive for degradation. One may speculate if the sequential phosphorylation constitutes a regulatory factor for receptor degradation, however, this needs further analysis to confirm. Endosomal phosphorylation of Eps15 and Hrs induce a shift in the membrane bound fractions of the respective proteins. FRAP analysis shows us that there is a significant redistribution of the mobile fraction, presumably to the cytosol (Figs 3a–d and 4a,b). Redistribution of the membrane bound fractions of Eps15 and Hrs proved to be a prerequisite for the degradation of EGF-R and PDGF-R. This suggests a mechanism where phosphorylated and ubiquinated Eps15 and Hrs are released to activate the sorting competent non-ubiquitinated/phosphorylated Hrs for further receptor sorting, supporting earlier work suggesting a redistribution from the endosomes to the cytosol upon receptor activation^21^. Immobile fraction relocation of Eps15 and Hrs proved to be functionally significant to ensure receptor degradation.

We have here shown that RTK activation induces a sequential phosphorylation of Eps15 and Hrs. This time dependent phosphorylation activates the translocation of Hrs and Eps15 from endosomes to cytosol. This particular translocation is dominant and specifically regulated by a redistribution of the immobile membrane bound fractions of Eps15 and Hrs. We have here identified an endosomal RTK ligand-induced mechanism that describes a new link to better understand the regulatory path of ligand activated receptors.

## Electronic supplementary material

Supplementary Movie 1

Supplementary Movie legends and supplementary table 1 and 2
