# Supplementary material for: Endosomal binding kinetics of Eps15 and Hrs specifically regulate the degradation of RTKs
Source: Sci Rep. 2017 Dec 21;7:17962. doi: 10.1038/s41598-017-17320-2 (PMC5740074; doi:10.1038/s41598-017-17320-2)
Supplement: Supplementary file 2 — Supplementary Movie legends and supplementary table 1 and 2 [file 41598_2017_17320_MOESM2_ESM.pdf]

Endosomal binding kinetics of Eps15 and Hrs specifically regulate the degradation of RTKs

Linda Hofstad Haugen[1], Frode Miltzow Skjeldal[1], Trygve Bergeland[1,2] and Oddmund Bakke[1*]

[1], Department of Biosciences, Centre of Immune Regulation, University of Oslo, Norway

[2] Present address: Kappa Bioscience AS, Oslo, Norway
[*] Corresponding author: Oddmund Bakke, oddmund.bakke@ibv.uio.no

Supplementary table legend

Supplementary table 1: FRAP data on single endosomes positive of Eps15-GFP, Hrs-YFP, Rab5mCherry or CtEEA1-GFP

Each dataset was fitted by non-linear regression, and the $T_{1/2}$ (seconds) and immobile fraction (IF) (%) is shown as mean values +/- standard deviation (s.d) of 10 independent measurements.

	N	EGF activation	$T_{1/2}$ (sec)	IF (%)
Eps15-GFP	10	0 min	1.97±0.06	21.4±0.9
Eps15-GFP	10	4 min	1.36±0.05	5.3±0.2
Eps15-GFP	10	8 min	1.51±0.09	13.2±0.8
Eps15-GFP	10	20 min	2.13±0.06	22.3±0.9
Eps15 Y850F-GFP	10	0 min	1.94±0.14	15.1±0.2
Eps15 Y850F-GFP	10	4 min	1.95±0.23	22.4±0.3
Eps15 Y850F-GFP	10	8 min	1.97±0.14	18.1±0.2
Eps15 Y850F-GFP	10	20 min	2.02±0.23	25.3±0.3
Hrs-YFP	10	0 min	6.22±1.38	45.4±0.5
Hrs-YFP	10	4 min	5.05±0.98	44.8±0.6
Hrs-YFP	10	8 min	2.99±0.28	27.9±0.2
Hrs-YFP	10	20 min	3.79±0.45	46.1±0.3
Hrs Y329F/Y334F-YFP	10	0 min	6.05±0.05	51.7±0.2
Hrs Y329F/Y334F-YFP	10	4 min	6.35±0.04	49.9±0.4
Hrs Y329F/Y334F-YFP	10	8 min	6.70±0.06	47.5±0.3
Hrs Y329F/Y334F-YFP	10	20 min	6.72±0.50	48.7±0.9
CtEEA1-GFP	10	0 min	4.70±0.19	33.8±3.4
CtEEA1-GFP	10	4 min	4.68±0.16	33.3±1.6
CtEEA1-GFP	10	8 min	4.70±0.20	32.5±3.0
CtEEA1-GFP	10	20 min	4.65±0.17	31.1±2.8
Rab5mCherry	10	0 min	22.85±0.29	33.5±5.1
Rab5mCherry	10	4 min	22.36±0.37	34.5±3.2
Rab5mCherry	10	8 min	22.65±0.42	35.9±4.8
Rab5mCherry	10	20 min	22.33±0.29	36.6±2.1

Supplementary table 2: FRAP data on single endosomes positive of Eps15-GFP or Hrs-YFP after PDGF stimulation

Each dataset was fitted by non-linear regression, and the $T_{1/2}$ (seconds) and immobile fraction (IF) (%) is shown as mean values +/- standard deviation (s.d) of 10 independent measurements.

	N	PDGF activation	$T_{1/2}$ (sec)	IF (%)
Eps15-GFP	10	0 min	2.38±0.15	18.7±0.2
Eps15-GFP	10	4 min	1.24±0.01	10.9±0.2
Eps15-GFP	10	8 min	1.70±0.08	15.2±0.1
Eps15-GFP	10	20 min	2.24±0.29	17.5±0.2
Eps15 Y850F-GFP	10	0 min	2.48±0.18	18.8±0.2
Eps15 Y850F-GFP	10	4 min	2.36±0.21	17.9±0.5
Eps15 Y850F-GFP	10	8 min	2.21±0.12	19.8±0.2
Eps15 Y850F-GFP	10	20 min	2.21±0.10	19.3±0.2
Hrs-YFP	10	0 min	5.44±0.34	44.8±0.3
Hrs-YFP	10	4 min	5.12±0.34	49.0±0.3
Hrs-YFP	10	8 min	2.87±0.24	36.9±0.3
Hrs-YFP	10	20 min	4.76±0.33	46.9±0.3
Hrs Y329F/Y334F-YFP	10	0 min	5.29±0.44	40.9±0.4
Hrs Y329F/Y334F-YFP	10	4 min	5.38v0.46	40.0±0.4
Hrs Y329F/Y334F-YFP	10	8 min	5.29±0.44	42.2±0.4
Hrs Y329F/Y334F-YFP	10	20 min	5.16±0.45	42.5±0.3

Supplementary Movie legends

Supplementary Movie 1:

This movie shows EGF-Alexa-647 internalization in M1 cells stably expressing Ii-pMep4 co-transfected with Eps15-GFP or Hrs-mRFP. EGF-Alexa-647 is internalized and transported to specific endosomal domains positive for Eps15-GFP or Hrs-mRFP on enlarged endosomes.

Supplementary Figures

Supplementary Figure 1; Western blot – Phosphorylation of Eps15-GFP after EGF stimulation (Figure 4C)

Gel number 1: The primary antibody used is anti-P-Tyrosine
Gel number 2: The primary antibody used is anti-GFP

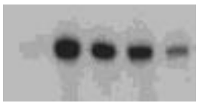

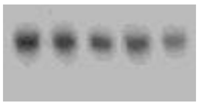

The samples in the two gels are:

1) Cells expressing Eps15-GFP and stimulated with EGF for 0 min.
2) Cells expressing Eps15-GFP and stimulated with EGF for 4 min.
3) Cells expressing Eps15-GFP and stimulated with EGF for 8 min.
4) Cells expressing Eps15-GFP and stimulated with EGF for 20 min.
5) Cells expressing Eps15-GFP and stimulated with EGF for 40 min.

Supplementary Figure 2; Western blot – Phosphorylation of Hrs-YFP after EGF stimulation (Figure 4C)

Gel number 1: The primary antibody used is anti-P-Tyrosine
Gel number 2: The primary antibody used is anti-GFP

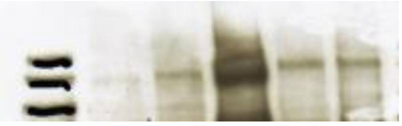

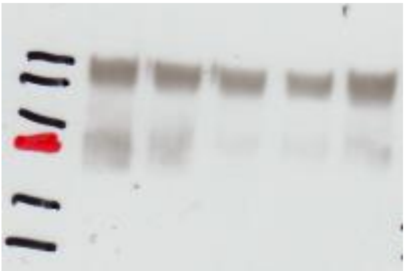

The samples in the two gels are:

1) Cells expressing Hrs-YFP and stimulated with EGF for 0 min.
2) Cells expressing Hrs-YFP and stimulated with EGF for 4 min.
3) Cells expressing Hrs-YFP and stimulated with EGF for 8 min.
4) Cells expressing Hrs-YFP and stimulated with EGF for 20 min.
5) Cells expressing Hrs-YFP and stimulated with EGF for 40 min.

Supplementary Figure 3; Western blot – Phosphorylation of Eps15-GFP after PDGF stimulation (Figure 4D)

Gel number 1: The primary antibody used is anti-P-Tyrosine
Gel number 2: The primary antibody used is anti-GFP

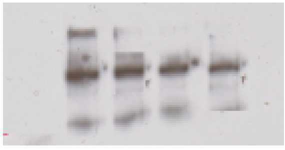

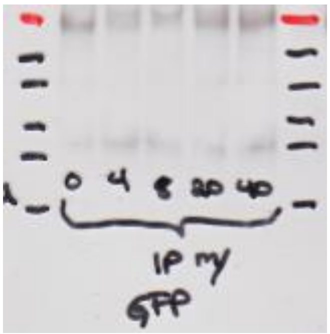

The samples in the two gels are:

1) Cells expressing Eps15-GFP and stimulated with PDGF for 0 min.
2) Cells expressing Eps15-GFP and stimulated with PDGF for 4 min.
3) Cells expressing Eps15-GFP and stimulated with PDGF for 8 min.
4) Cells expressing Eps15-GFP and stimulated with PDGF for 20 min.
5) Cells expressing Eps15-GFP and stimulated with PDGF for 40 min.

Supplementary Figure 4; Western blot – Phosphorylation of Hrs-YFP after PDGF stimulation (Figure 4D)

Gel number 1: The primary antibody used is anti-P-Tyrosine
Gel number 2: The primary antibody used is anti-GFP

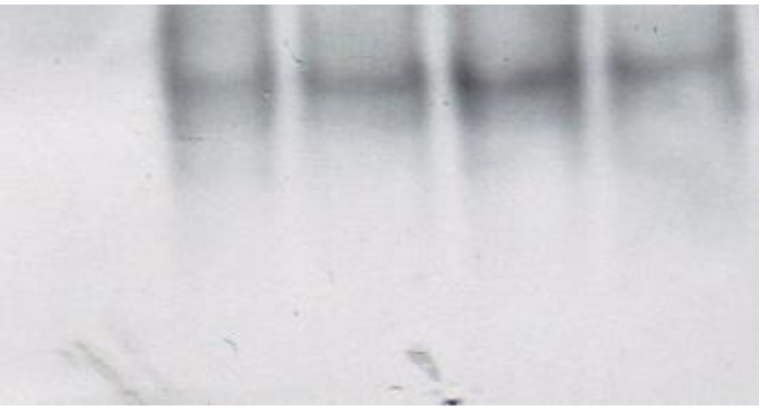

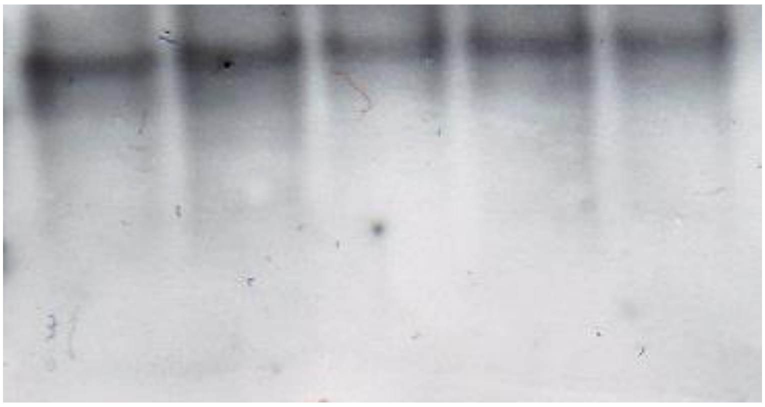

The samples in the two gels are:

1) Cells expressing Hrs-YFP and stimulated with PDGF for 0 min.
2) Cells expressing Hrs-YFP and stimulated with PDGF for 4 min.
3) Cells expressing Hrs-YFP and stimulated with PDGF for 8 min.
4) Cells expressing Hrs-YFP and stimulated with PDGF for 20 min.
5) Cells expressing Hrs-YFP and stimulated with PDGF for 40 min.

Supplementary Figure 5; Western blot – EGF stimulation of Eps15Y850F-GFP (6C)

Gel number 1: The primary antibody used is anti-Eps15
Gel number 2: The primary antibody used is anti-GFP
Gel number 3: The primary antibody used is anti-P-Tyrosine

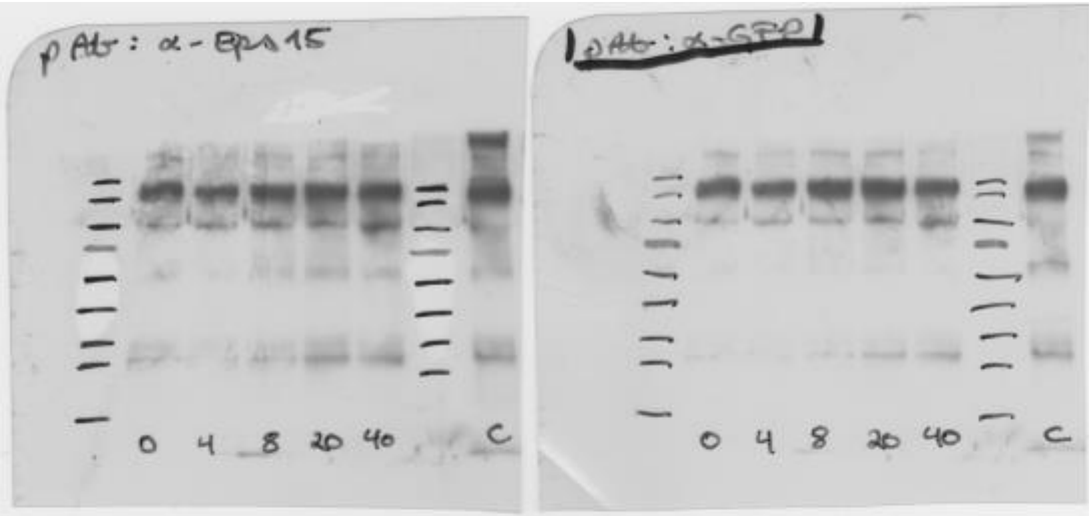

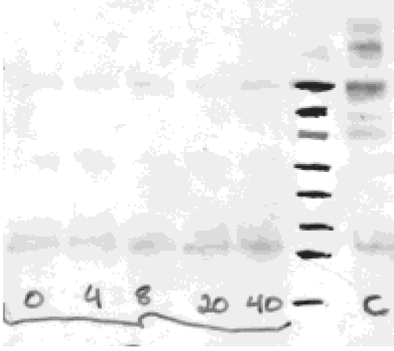

The samples in the three gels are:

1) Cells expressing Eps15Y850F-GFP and stimulated with EGF for 0 min.
2) Cells expressing Eps15Y850F-GFP and stimulated with EGF for 4 min.
3) Cells expressing Eps15Y850F-GFP and stimulated with EGF for 8 min.
4) Cells expressing Eps15Y850F-GFP and stimulated with EGF for 20 min.
5) Cells expressing Eps15Y850F-GFP and stimulated with EGF for 40 min.
6) Standard
7) Control: Cells expressing Eps15-GFP (wt) and stimulated with EGF for 4 min.

Supplementary Figure 6; Western blot – EGF stimulation of HrsY329, 334F-YFP (6C)

Gel number 1: The primary antibody used is anti-P-Tyrosine
Gel number 2: The primary antibody used is anti-Hrs
Gel number 3: The primary antibody used is anti-GFP

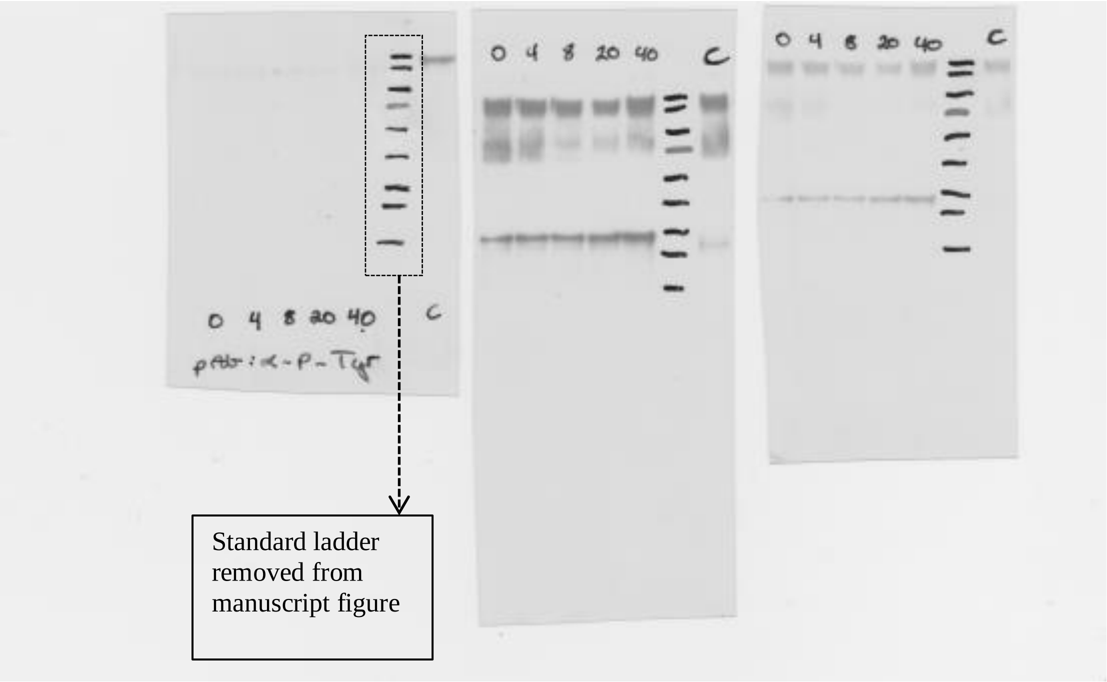

The samples in the three gels are:

1) Cells expressing HrsY329, 334F-YFP and stimulated with EGF for 0 min.
2) Cells expressing HrsY329, 334F-YFP and stimulated with EGF for 4 min.
3) Cells expressing HrsY329, 334F-YFP and stimulated with EGF for 8 min.
4) Cells expressing HrsY329, 334F-YFP and stimulated with EGF for 20 min.
5) Cells expressing HrsY329, 334F-YFP and stimulated with EGF for 40 min.
6) Standard
7) Control: Cells expressing Hrs-YFP (wt) and stimulated with EGF for 8 min.

In figure 6C in we have removed the standard ladder and merged the control line with the rest of the blot (see the original data above).

Supplementary Figure 7; Degradation assay – Eps15-GFP (Figure 7A)

<u>EGFR degradation assay - Eps15-GFP:</u>

Gel number 1: The primary antibody used is anti-EGFR
Gel number 2: The primary antibody used is anti-Eps15
Gel number 3: The primary antibody used is anti-GFP

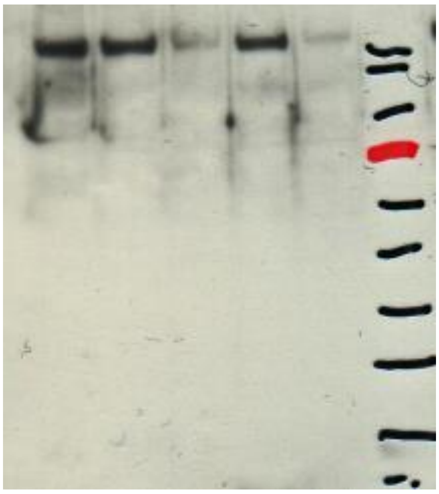

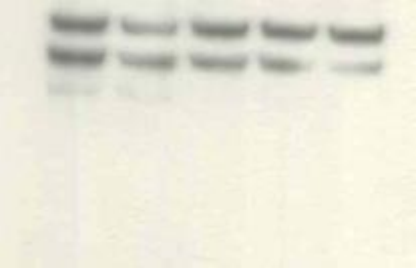

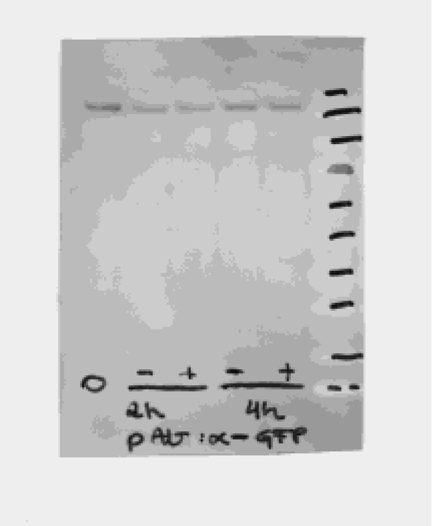

The samples in the three gels are:

1) Cells expressing Eps15-GFP (control)
2) Cells expressing Eps15-GFP; Treated with CHX for 2h
3) Cells expressing Eps15-GFP; Treated with CHX for 2h and stimulated with EGF for 2 min.
4) Cells expressing Eps15-GFP; Treated with CHX for 4h
5) Cells expressing Eps15-GFP; Treated with CHX for 4h and stimulated with EGF for 4h.

PDGFR degradation assay - Eps15-GFP:

Gel number 1: The primary antibody used is anti- PDGFR
Gel number 2: The primary antibody used is anti-Eps15
Gel number 3: The primary antibody used is anti-GFP

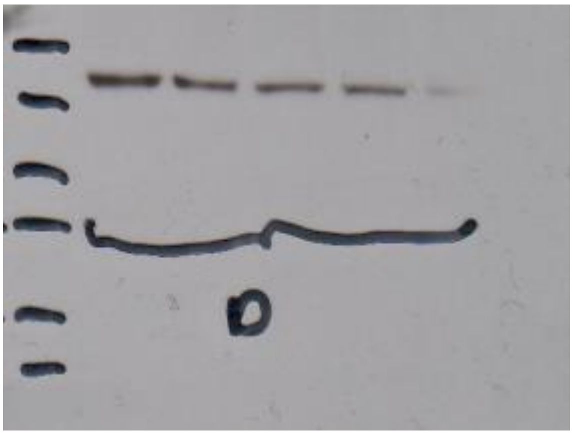

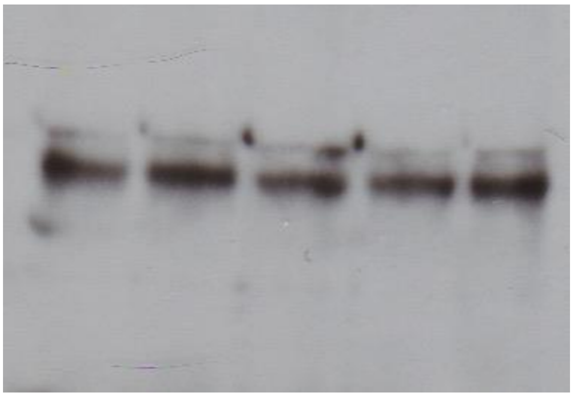

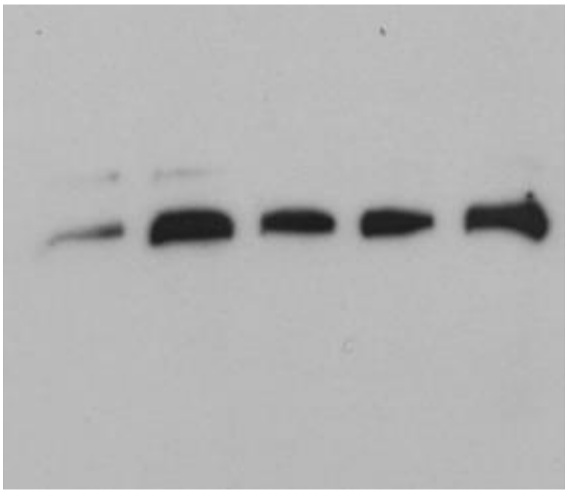

The samples in the three gels are:

1) Cells expressing Eps15-GFP (control)
2) Cells expressing Eps15-GFP; Treated with CHX for 2h
3) Cells expressing Eps15-GFP; Treated with CHX for 2h and stimulated with PDGF for 2 min.
4) Cells expressing Eps15-GFP; Treated with CHX for 4h

5) Cells expressing Eps15-GFP; Treated with CHX for 4h and stimulated with PDGF for 4h.

Supplementary Figure 8; Degradation assay – Hrs-YFP (Figure 7B)

EGFR degradation assay – Hrs-YFP:

Gel number 1: The primary antibody used is anti-EGFR
Gel number 2: The primary antibody used is anti-Hrs
Gel number 3: The primary antibody used is anti-GFP

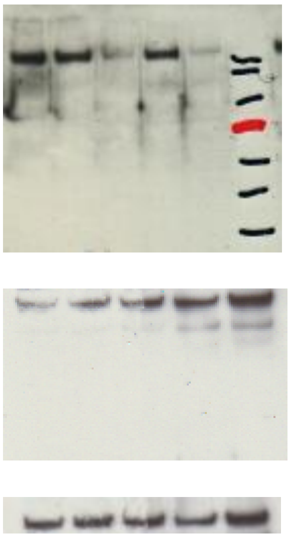

The samples in the three gels are:

1) Cells expressing Hrs-YFP (control)
2) Cells expressing Hrs-YFP; Treated with CHX for 2h
3) Cells expressing Hrs-YFP; Treated with CHX for 2h and stimulated with EGF for 2 min.
4) Cells expressing Hrs-YFP; Treated with CHX for 4h
5) Cells expressing Hrs-YFP; Treated with CHX for 4h and stimulated with EGF for 4h.

PDGFR degradation assay – Hrs-YFP:

Gel number 1: The primary antibody used is anti-PDGFR
Gel number 2: The primary antibody used is anti-Hrs
Gel number 3: The primary antibody used is anti-GFP

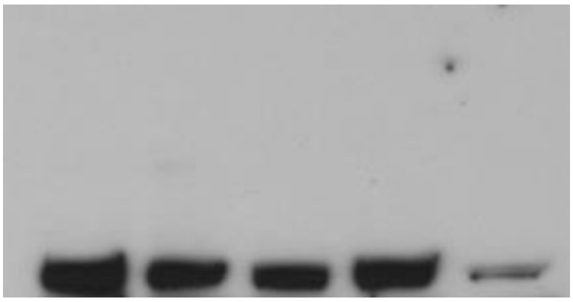

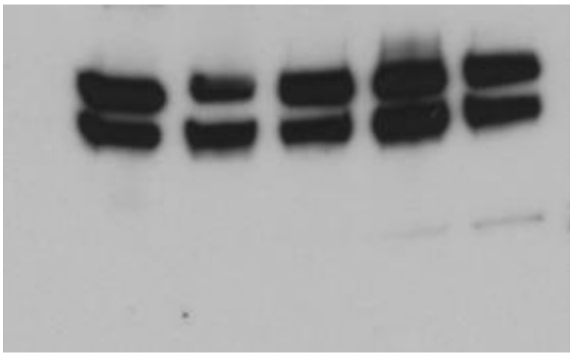

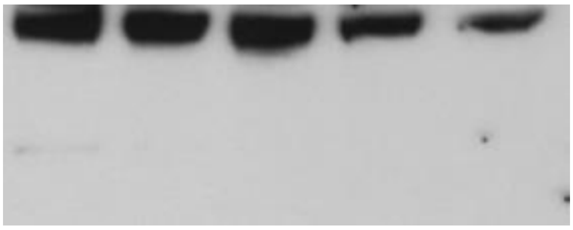

The samples in the three gels are:

1) Cells expressing Hrs-YFP (control)
2) Cells expressing Hrs-YFP; Treated with CHX for 2h
3) Cells expressing Hrs-YFP; Treated with CHX for 2h and stimulated with PDGF for 2 min.
4) Cells expressing Hrs-YFP; Treated with CHX for 4h
5) Cells expressing Hrs-YFP; Treated with CHX for 4h and stimulated with PDGF for 4h.

Supplementary Figure 9; Degradation assay – Eps15Y850F-GFP (Figure 7C)

<u>EGFR degradation assay – Eps15Y850F:</u>

Gel number 1: The primary antibody used is anti-EGFR
Gel number 2: The primary antibody used is anti-Eps15
Gel number 3: The primary antibody used is anti-GFP

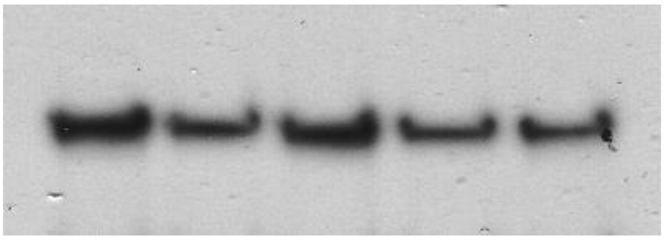

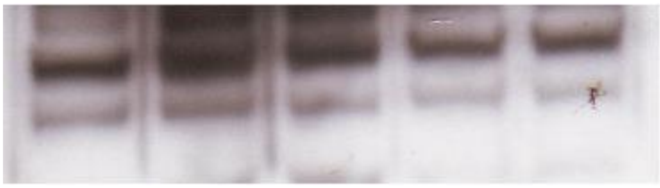

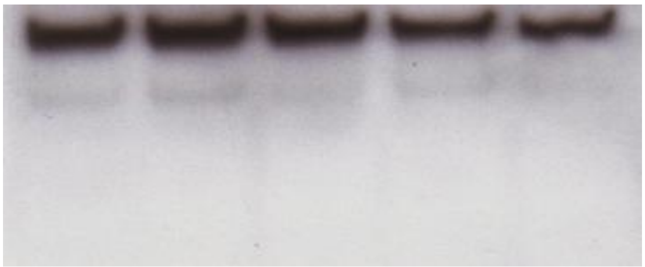

The samples in the three gels are:

1) Cells expressing Eps15Y850F-GFP (control)
2) Cells expressing Eps15Y850F-GFP; Treated with CHX for 2h
3) Cells expressing Eps15Y850F-GFP; Treated with CHX for 2h and stimulated with EGF for 2 min.
4) Cells expressing Eps15Y850F-GFP; Treated with CHX for 4h
5) Cells expressing Eps15Y850F-GFP; Treated with CHX for 4h and stimulated with EGF for 4h.

PDGFR degradation assay – Eps15Y850F:

Gel number 1: The primary antibody used is anti-PDGFR
Gel number 2: The primary antibody used is anti-Eps15
Gel number 3: The primary antibody used is anti-GFP

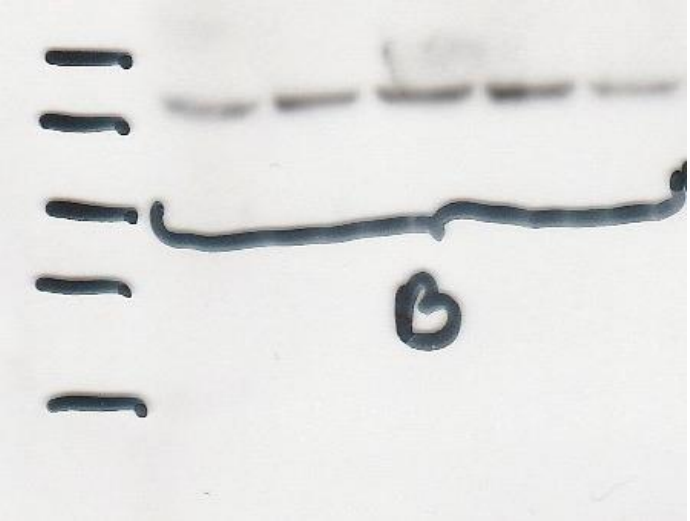

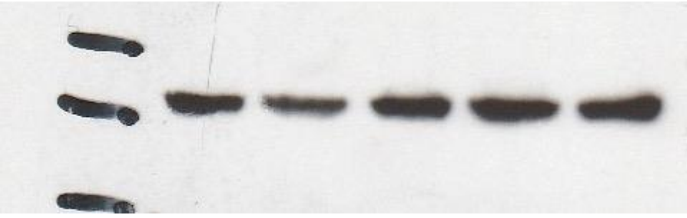

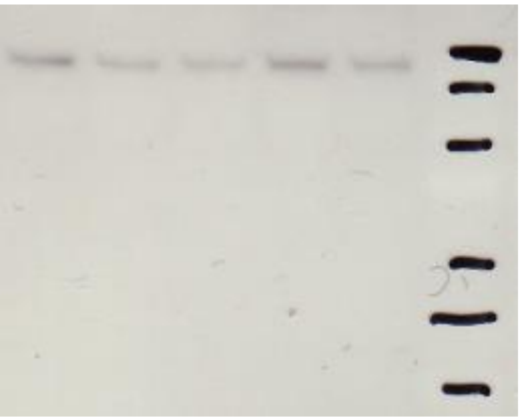

The samples in the three gels are:

1) Cells expressing Eps15Y850F-GFP (control)
2) Cells expressing Eps15Y850F-GFP; Treated with CHX for 2h
3) Cells expressing Eps15Y850F-GFP; Treated with CHX for 2h and stimulated with PDGF for 2 min.
4) Cells expressing Eps15Y850F-GFP; Treated with CHX for 4h
5) Cells expressing Eps15Y850F-GFP; Treated with CHX for 4h and stimulated with PDGF for 4h.

Supplementary Figure 10; Degradation assay – HrsY329, 344F-YFP (Figure 7D)

<u>EGFR degradation - HrsY29, 334F-GFP;</u>

Gel number 1: The primary antibody used is anti-EGFR

Gel number 2: The primary antibody used is anti-Hrs
Gel number 3: The primary antibody used is anti-GFP

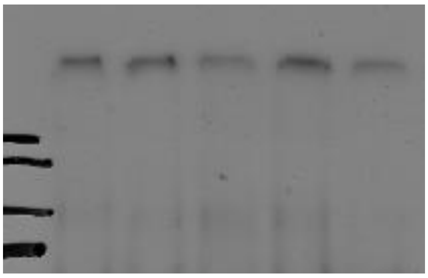

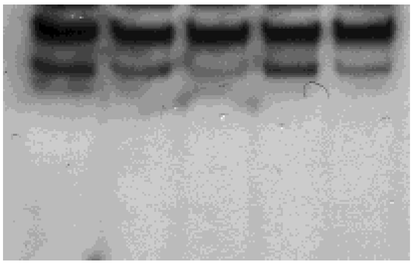

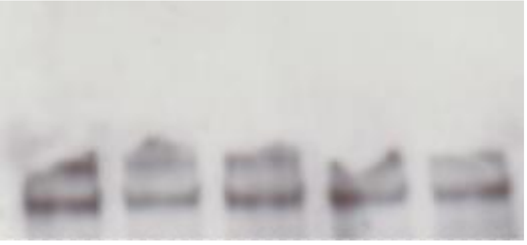

The samples in the three gels are:

1) Cells expressing HrsY29, 334F-GFP (control)
2) Cells expressing HrsY29, 334F-GFP; Treated with CHX for 2h
3) Cells expressing HrsY29, 334F-GFP; Treated with CHX for 2h and stimulated with EGF for 2 min.
4) Cells expressing HrsY29, 334F-GFP; Treated with CHX for 4h
5) Cells expressing HrsY29, 334F-GFP; Treated with CHX for 4h and stimulated with EGF for 4h.

<u>PDGFR degradation - HrsY29, 334F-GFP;</u>

Gel number 1: The primary antibody used is anti-PDGFR
Gel number 2: The primary antibody used is anti-Hrs
Gel number 3: The primary antibody used is anti-GFP

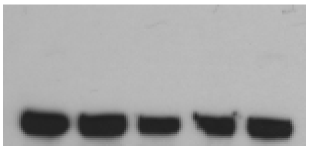

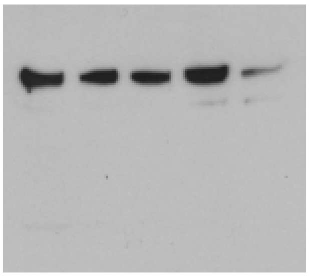

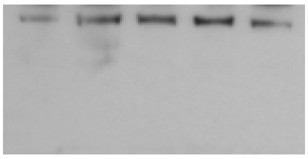

The samples in the three gels are:

1) Cells expressing HrsY29, 334F-GFP (control)
2) Cells expressing HrsY29, 334F-GFP; Treated with CHX for 2h
3) Cells expressing HrsY29, 334F-GFP; Treated with CHX for 2h and stimulated with PDGF for 2 min.
4) Cells expressing HrsY29, 334F-GFP; Treated with CHX for 4h
5) Cells expressing HrsY29, 334F-GFP; Treated with CHX for 4h and stimulated with PDGF for 4h.
